# Ccl5 establishes an autocrine high-grade glioma growth regulatory circuit critical for mesenchymal glioblastoma survival

**DOI:** 10.18632/oncotarget.16516

**Published:** 2017-03-23

**Authors:** Yuan Pan, Laura J. Smithson, Yu Ma, Dolores Hambardzumyan, David H. Gutmann

**Affiliations:** ^1^ Department of Neurology, Washington University School of Medicine, St. Louis, MO, USA; ^2^ Department of Oncology, Aflac Cancer and Blood Disorders Center, Emory University, GA, USA

**Keywords:** chemokine, glioma, neurofibromin, mTOR, CD44

## Abstract

Glioblastoma (GBM) is the most common malignant brain tumor in adults, with a median survival of 15 months. These poor clinical outcomes have prompted the development of drugs that block neoplastic cancer cell growth; however, non-neoplastic cell-derived signals (chemokines and cytokines) in the tumor microenvironment may also represent viable treatment targets. One such chemokine, Ccl5, produced by low-grade tumor-associated microglia, is responsible for maintaining neurofibromatosis type 1 (NF1) mouse optic glioma growth *in vivo*. Since malignant gliomas may achieve partial independence from growth regulatory factors produced by non-neoplastic cells in the tumor microenvironment by producing the same cytokines secreted by the stromal cells in their low-grade counterparts, we tested the hypothesis that CCL5/CCL5-receptor signaling in glioblastoma creates an autocrine circuit important for high-grade glioma growth. Herein, we demonstrate that increased *CCL5* expression was restricted to both human and mouse mesenchymal GBM (M-GBM), a molecular subtype characterized by *NF1* loss. We further show that the *NF1* protein, neurofibromin, negatively regulates Ccl5 expression through suppression of AKT/mTOR signaling. Consistent with its role as a glioblastoma growth regulator, Ccl5 knockdown in M-GBM cells reduces M-GBM cell survival *in vitro*, and increases mouse glioblastoma survival *in vivo*. Finally, we demonstrate that Ccl5 operates through an unconventional CCL5 receptor, CD44, to inhibit M-GBM apoptosis. Collectively, these findings reveal an *NF1*-dependent CCL5-mediated pathway that regulates M-GBM cell survival, and support the concept that paracrine factors important for low-grade glioma growth can be usurped by high-grade tumors to create autocrine regulatory circuits that maintain malignant glioma survival.

## INTRODUCTION

Malignant gliomas (glioblastoma or GBM) are the most common brain tumor in adults, with approximately over 13,000 individuals in the United States dying from their cancer each year. After initial diagnosis, the median survival is only 9 months [1, 2], which improves to ~15 months following surgical resection, radiation and chemotherapy [3]. In an effort to improve clinical outcomes for people with this deadly cancer, numerous conventional and biologically-based anti-neoplastic treatments have been evaluated that specifically target the rapidly dividing cancer cells. Unfortunately, these newer therapies have resulted in only modest increases in overall survival, highlighting the urgent need to identify more effective therapies for these brain malignancies.

Over the past decades, numerous reports have described an instructive role for the tumor microenvironment in glioma formation and maintenance [4]. In this regard, glioblastomas contain a rich non-neoplastic component, including endothelial cells and immune system cells (e.g., microglia and macrophages), whose function is dictated by small molecules responsible for their recruitment and activation. One class of these proteins, chemokines, is critical for microglia and macrophage infiltration and activation through the expression of chemokine receptors on their plasma membranes. Consistent with an ecosystem paradigm in which non-neoplastic cells attracted by cancer cells in turn support neoplastic cell growth, inhibition of chemokine expression or chemokine receptor function has been shown to attenuate both low-grade and high-grade glioma growth in numerous experimental preclinical model systems *in vivo*. For example, CCL2 recruits regulatory T cells and myeloid-derived suppressor cells to increase GBM growth [5], as well as indirectly increases glioma invasiveness by stimulating IL-6 secretion from tumor-associated microglia (TAM) [6]. In addition, CXCL12 and its receptors CXCR4/CXCR7 function by maintaining GBM stem-like cell survival and increasing glioma cell invasion [7, 8]. Similarly, colony stimulating factor-1 (CSF-1) creates a supportive glioma microenvironment, such that silencing CSF-1-receptor signaling on macrophages is sufficient to reduce high-grade glioma growth in mice [9, 10]. Together, these experimental observations establish a fundamental role for chemokines in glioma biology.

In addition to the chemokines mentioned above, we have recently discovered that Ccl5 produced by tumor-associated microglia is a potent paracrine regulator of neurofibromatosis type 1 (NF1)-associated murine low-grade optic glioma growth [11]. In this experimental system, Ccl5 was sufficient to stimulate *Nf1*-deficient optic nerve glial cell proliferation *in vitro*, whereas Ccl5 antibody-mediated inhibition attenuated mouse optic glioma proliferation *in vivo*. Interestingly, CCL5 has recently emerged as a key member of an autocrine cytokine circuit critical for KRAS-driven lung cancer growth [12]. Since the *NF1* protein, neurofibromin, functions as a negative regulator of RAS, specifically K-RAS in astroglial cells [13, 14], and *NF1* mutation/loss characterizes the mesenchymal GBM (M-GBM) molecular subtype [15], we sought to determine whether Ccl5 expression creates a unique autocrine circuit for mesenchymal glioblastoma growth.

In this report, we describe for the first time, that increased *CCL5* expression is enriched in both human and mouse M-GBM with *NF1* loss. Leveraging *Nf1*-deficient murine M-GBM models, we further demonstrate that Ccl5 shRNA-mediated knockdown increases glioblastoma cell apoptosis *in vitro*, as well as extends mouse glioblastoma survival *in vivo*. Moreover, neurofibromin negatively regulates Ccl5 expression through RAS/AKT/mTOR pathway activation. Lastly, Ccl5 produced by the M-GBM cells acts in a cell autonomous fashion to increase M-GBM cell survival using a non-conventional CCL5 receptor, CD44, to suppress programmed cell death. Collectively, these findings broaden the concept that growth regulatory factors produced by non-neoplastic cells in the low-grade glioma microenvironment are employed to establish autocrine circuits in high-grade gliomas, thus facilitating a state of relative stromal independence.

## RESULTS

### Ccl5 functions in a cell-autonomous fashion to increase M-GBM survival

**Figure 1:**
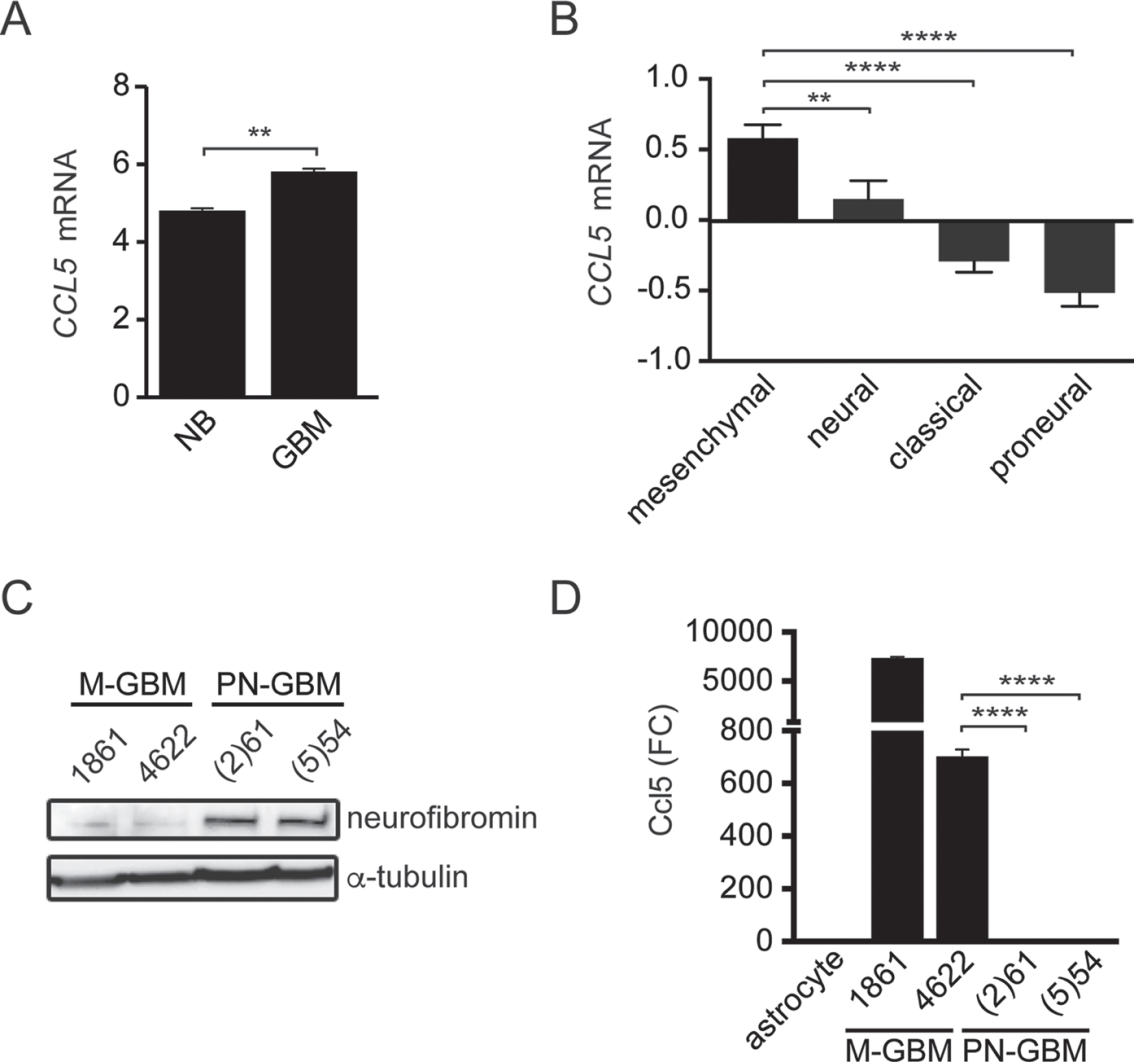
M-GBM express high levels of CCL5 (**A**) Human GBM samples (GSE16011 [16]) have elevated *CCL5* mRNA levels relative to normal brain (NB). **p = 0.0023. (**B**) *CCL5* mRNA expression (Log2 Z-score) in TCGA GBM (provisional) samples segregated by molecular subgroup reveals that mesenchymal and proneural GBM express the highest and lowest *CCL5* levels, respectively. **p = 0.0095; ****p < 0.0001. *n* = 106, 55, 102, and 96 for the mesenchymal, neural, classical and proneural molecular GBM subgroups, respectively. (**C**) M-GBM cells are neurofibromin deficient, while PN-GBM cells express neurofibromin. (**D**) Ccl5 ELISA analysis of the culture medium reveals higher levels of Ccl5 in M-GBM cells relative to PN-GBM cells and primary astrocytes. ****p < 0.0001. FC, fold change.

Based on our previous studies examining CCL5 expression and function in low-grade glioma, we first sought to determine whether CCL5 expression was higher in human GBM tumors relative to non-neoplastic brain. Using the GSE16011 [16] dataset, for which normal brain was available for comparison, *CCL5* mRNA levels were higher in GBM samples relative to their non-neoplastic counterpart (Figure 1A). We next analyzed *CCL5* mRNA expression in the four GBM molecular subtypes using data from the Cancer Genome Atlas (TCGA, provisional), and found that mesenchymal GBM (M-GBM) exhibited the highest levels of *CCL5* mRNA expression, while proneural GBM (PN-GBM) expressed the lowest (Figure 1B).

This relative enrichment of *CCL5* expression in the mesenchymal subtype, which frequently exhibits loss of function mutations in the *NF1* tumor suppressor gene [15], prompted us to focus on murine models characterized by *Nf1* loss. Using two representative mouse M-GBM cell lines (1861 and 4622), which lack *Nf1* and *Tp53* expression [17], and two representative mouse proneural GBM cell lines ((2)61 and (5)54), which retain neurofibromin expression (Figure 1C) [18], we found that Ccl5 levels were elevated in the culture medium of mouse M-GBM cells relative to PN-GBM cells, as well as to wild-type primary mouse astrocytes (Figure 1D).

**Figure 2:**
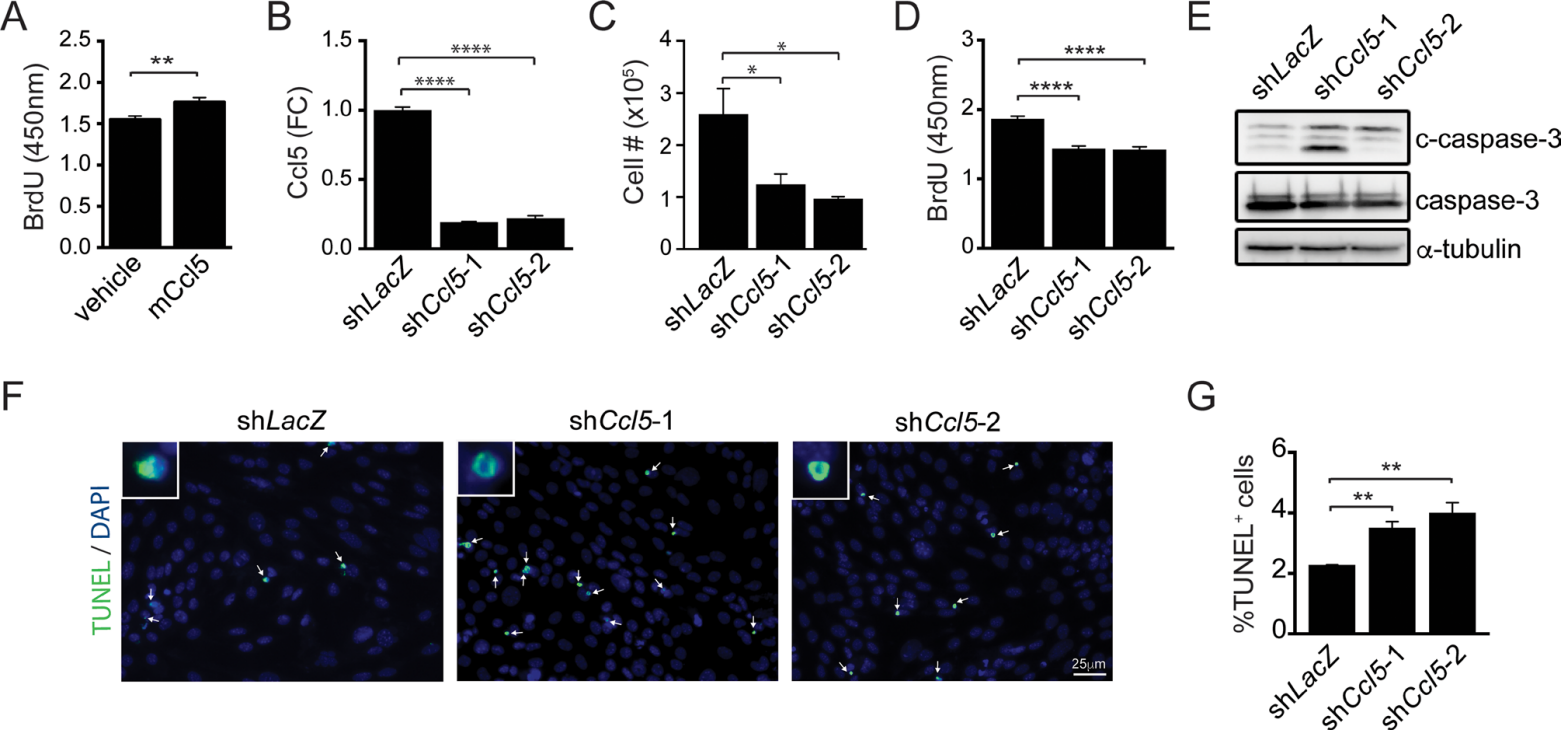
Ccl5 functions in a cell-autonomous fashion to increase M-GBM cell survival (**A**) Increased BrdU incorporation was observed in 1861 cells following treatment with mouse Ccl5 (mCcl5). Vehicle was 0.5% BSA in PBS. **p = 0.009. (**B**) ELISA analysis of 1861 cell medium shows that *Ccl5* KD (sh*Ccl5*-1, sh*Ccl5*-2) cells produce less Ccl5 compared to control (sh*LacZ*) cells. ****p < 0.0001. FC, fold change. (**C**) *Ccl5* KD cells exhibit reduced cell numbers. *p = 0.0428 (sh*LacZ* vs sh*Ccl5*-1), and *p* = 0.0162 (sh*LacZ* vs sh*Ccl5*-2). (**D**) *Ccl5* KD cells exhibit reduced cell growth (BrdU incorporation). ****p < 0.0001. (**E**) Western blotting demonstrates increased cleaved caspase-3 (c-caspase-3) expression following *Ccl5* KD. (**F**, **G**) *Ccl5* KD increases the percentage of TUNEL^+^ cells (green; arrows). DAPI-stained nuclei (inset) are shown in blue. **p = 0.0039 (sh*LacZ* vs sh*Ccl5*-1), and *p* = 0.0063 (sh*LacZ* vs sh*Ccl5*-2). Scale bar, 25 μm.

To determine whether M-GBM cell growth was dependent on Ccl5 expression, 1861 (M-GBM) cells were grown in the presence of mouse Ccl5 (mCcl5). mCcl5 treatment increased cell growth, as revealed by ~14% increase in BrdU incorporation relative to the vehicle treatment (Figure 2A). Next, we reduced *Ccl5* levels in 1861 cells by shRNA-mediated knockdown (KD). Using two independent constructs (sh*Ccl5*-1 and sh*Ccl5*-2), Ccl5 protein levels were reduced to ~20% of control levels (*LacZ* shRNA; Figure 2B), resulting in reduction in cell number (Figure 2C) and cell growth (BrdU incorporation; Figure 2D) as a consequence of increased apoptosis (increased cleaved caspase-3 expression and %TUNEL^+^ cells; Figure 2E–2G). Similar results were obtained using another M-GBM cell line (4622; Supplementary Figure 1A–1E). Importantly, treating PN-GBM cells with mCcl5 did not affect cell growth, suggesting that Ccl5 is not a regulator for PN-GBM growth (Supplementary Figure 1F).

**Figure 3:**
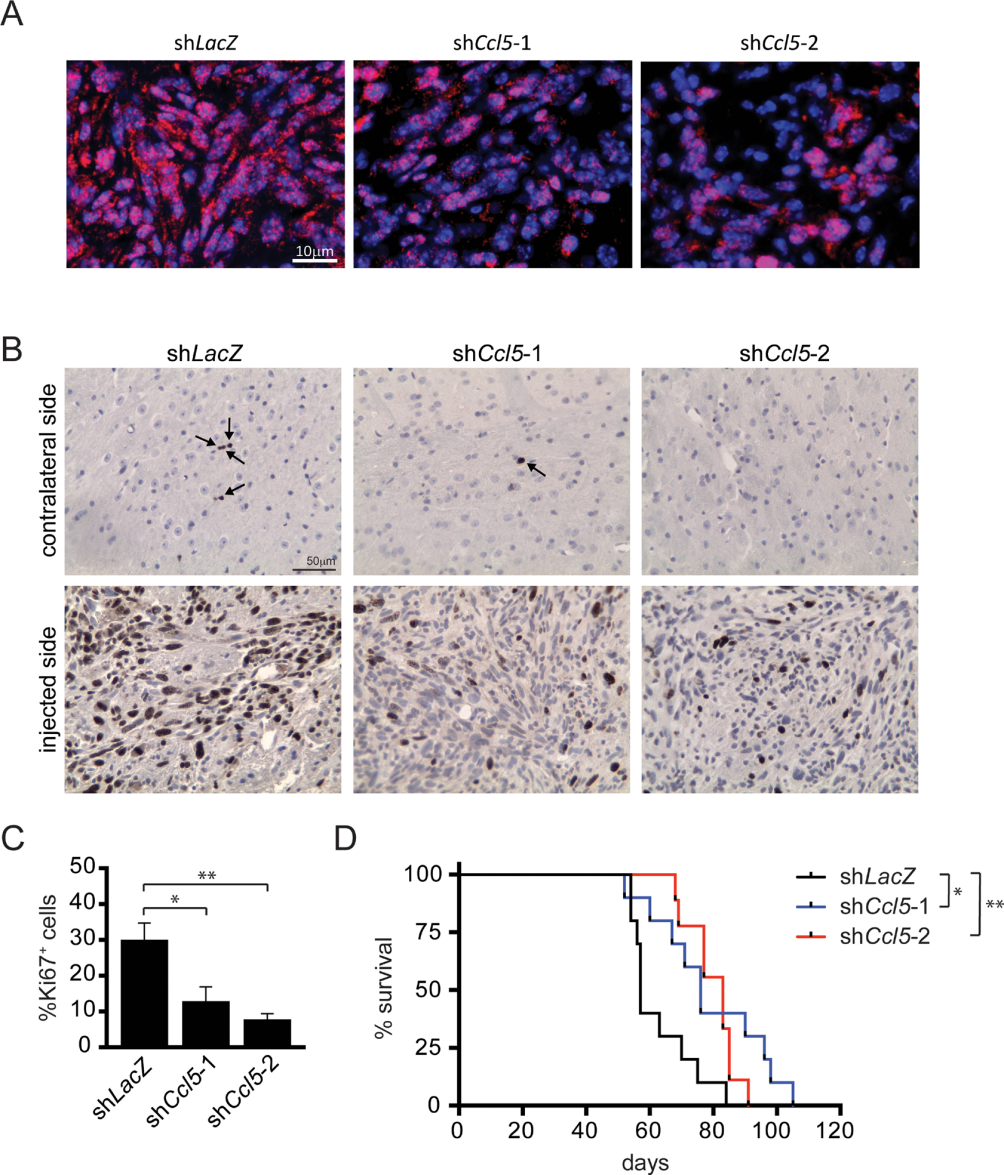
*Ccl5* knockdown reduces M-GBM growth and prolongs mouse survival *in vivo* *Ccl5* KD (sh*Ccl5*-1, sh*Ccl5*-2) and control (sh*LacZ*) 1861 cells were implanted into the striata of C57BL/6 mice. *Ccl5* KD groups have fewer Ccl5^+^ (red; A), Ki67^+^ cells (**B**, **C**, arrow) relative to controls. *p = 0.0145; **p = 0.0014. Scale bars, 10 μm (**A**) and 50 μm (B). (**D**) Kaplan-Meier survival curves demonstrate that the *Ccl5* KD groups have prolonged survival relative to controls. Each group contains 9–10 mice. *p = 0.0117; **p = 0.0037 (Log-rank test).

Based on these *in vitro* results, we also sought to determine whether Ccl5 is required for M-GBM growth *in vivo*. Control (sh*LacZ*) and *Ccl5* KD (sh*Ccl5*-1 and sh*Ccl5*-2) 1861 cells were stereotactically implanted into the striata of 4-week-old wild-type C57BL/6 mice. Both *Ccl5* KD groups demonstrated reduced Ccl5 levels (Figure 3A), reduced cell proliferation (%Ki67^+^ cells, Figure 3B, 3C) at time of death, as well as longer survival relative to the control (sh*LacZ*) group (Figure 3D). In addition, tumor implants dissected at 6-weeks post-implantation demonstrated reduced Ccl5 levels, increased apoptosis (cleaved caspase-3 expression), and reduced cell proliferation (PCNA expression) in the *Ccl5* KD groups (Supplementary Figure 2).

Given the role of Ccl5 as a chemokine, it may recruit monocytes to the implantation site to facilitate tumor growth; however, we found no change in the number of recruited monocytes or chemokine expression within the tumor between the control and *Ccl5* KD groups (data not shown). In addition, we sought to determine whether Ccl5 produced by non-neoplastic cells contributes to M-GBM tumor growth by comparing M-GBM cell growth in wild-type versus *Ccl5*−/− mouse striata. Mouse survival and tumor proliferation were similar between the two groups, arguing against a major stromal source for Ccl5 in mediating the observed M-GBM survival effects (Supplementary Figure 3). Collectively, these observations demonstrate that Ccl5 positively regulates M-GBM growth *in vivo* and *in vitro* through an autocrine stimulatory mechanism.

### Neurofibromin negatively regulates Ccl5 expression through suppression of AKT/mTOR pathway activation

**Figure 4:**
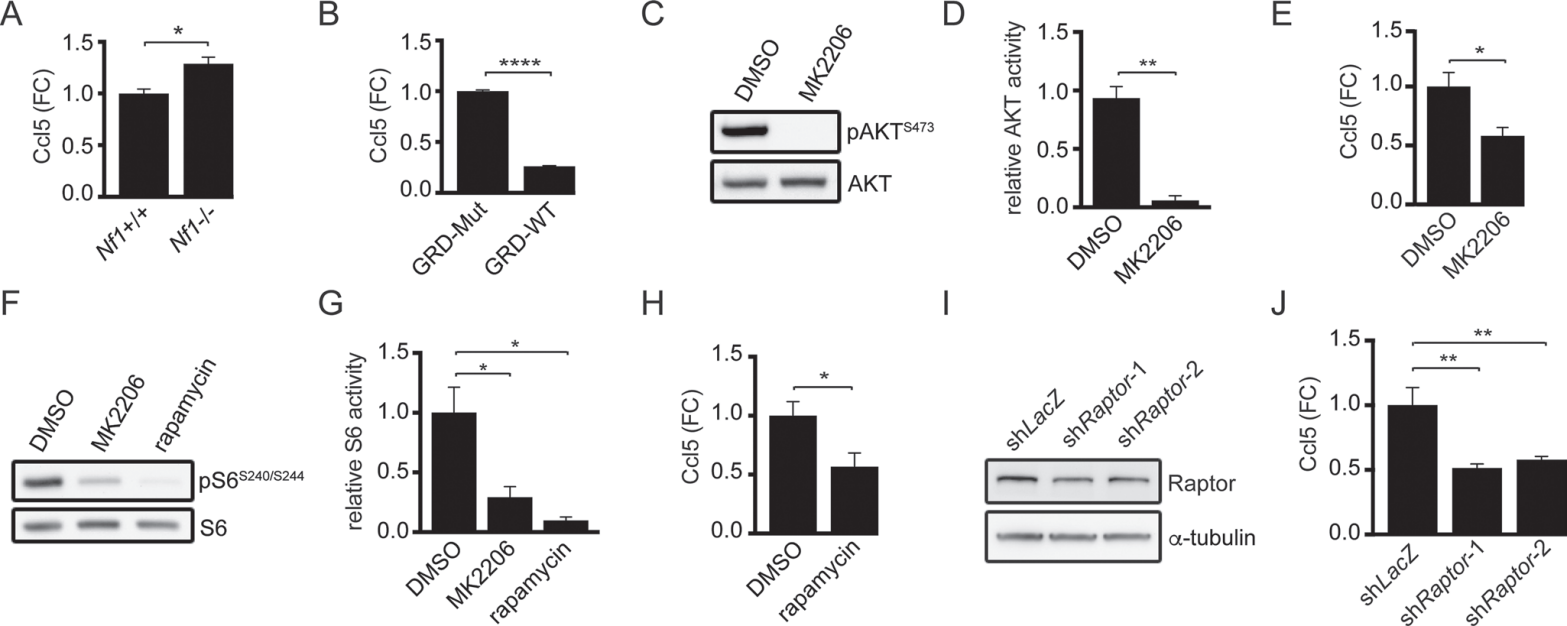
Ccl5 expression in M-GBM cells is regulated by neurofibromin-mediated AKT/mTOR pathway suppression (**A**) ELISA analysis demonstrates greater Ccl5 levels in the medium from *Nf1*−/− astrocytes relative to their wild-type (*Nf1*+/+) counterparts. *p = 0.01. FC, fold change. (**B**) Wild-type NF1-GRD (GRD-WT) expression in 1861 cells reduced Ccl5 expression relative to the NF1-GRD R1276P mutant (GRD-Mut). ****p < 0.0001. (**C**, **D**) Treatment of 1861 cells with MK2206 (AKT inhibitor) reduced AKT activity (phospho-AKT^S473^/AKT ratio) compared to DMSO (vehicle control). **p = 0.0012. (**E**) ELISA reveals reduced Ccl5 expression following MK2206 treatment. *p = 0.0258. FC, fold change. (**F**, **G**) MK2206 or rapamycin (mTOR inhibitor) treatment reduced mTOR activity (phospho-S6^S240/S244^/S6 ratio). *p = 0.0362 (DMSO vs MK2206) and *p* = 0.0116 (DMSO vs rapamycin). (**H**) Rapamycin-treated 1861 cells had reduced Ccl5 levels in the medium compared to DMSO controls. *p = 0.0313. FC, fold change. (**I**) Western blotting demonstrates that shRNA-mediated *Raptor* KD (sh*Raptor*-1 and sh*Raptor*-2) reduced Raptor protein expression in 1861 cells relative to control (sh*LacZ*) vector. (**J**) *Raptor* KD results in reduced Ccl5 levels in the medium. **p = 0.002 (sh*LacZ* vs sh*Raptor*-1), and *p* = 0.0034 (sh*LacZ* vs sh*Raptor*-2). FC, fold change.

Since increased *CCL5* expression predominates in the M-GBM molecular subtype and mouse *Nf1*-deficient GBM cells exhibit reduced growth following *Ccl5* KD, we tested the hypothesis that the *NF1* protein (neurofibromin) function is responsible for regulating Ccl5 expression. Using primary brainstem astrocytes from postnatal day 1 mice (*Nf1*^flox/flox^ astrocytes infected with Ad5-LacZ virus, *Nf1+/+*; *Nf1*^flox/flox^ astrocytes infected with Ad5-Cre virus, *Nf1*−/−) (Supplementary Figure 4A), Ccl5 levels in the medium were increased in *Nf1*−/− astrocytes relative to wild-type (*Nf1+/+*) controls (Figure 4A). To establish causality, we re-introduced the functional domain of neurofibromin (NF1-RAS GTPase-activating protein regulatory domain [NF1-GRD; GRD-WT]; Supplementary Figure 4B). A mutant NF1-GRD construct (NF1-GRD^R1276P^; GRD-Mut) defective at suppressing RAS activity was used as a control for RAS-GAP activity [19]. Expression of WT, but not mutant, NF1-GRD reduced Ccl5 levels in 1861 cells (Figure 4B), demonstrating that neurofibromin negatively regulates Ccl5 expression.

Neurofibromin regulates glioma cell growth through the AKT/mTOR pathway [20, 21], such that *Nf1*−/− astrocytes exhibit AKT/mTOR hyperactivation relative to *Nf1*+/+ (wild-type) astrocytes (Supplementary Figure 4A). Consistent with this neurofibromin regulatory function, AKT/mTOR activity in 1861 cells was reduced following NF1-GRD expression (Supplementary Figure 4C). Moreover, AKT pharmacological inhibition (MK2206) of M-GBM cells similarly reduced AKT activity (Figure 4C, 4D) and Ccl5 expression (Figure 4E). In addition, AKT inhibition in 1861 cells reduced mTOR activity (Figure 4F, 4G), and mTOR inhibition using rapamycin (Figure 4F, 4G) reduced Ccl5 expression (Figure 4H). The concentrations and durations of inhibitor treatments were specifically selected so that cell growth was not affected (Supplementary Figure 4D), arguing that any potential changes in Ccl5 expression were a secondary effect of suppressed cell growth. As a complementary approach, we employed shRNA knockdown of Raptor, a molecule critical for the function of mTOR complex-1 (Figure 4I). Similar to rapamycin treatment, *Raptor* KD reduced Ccl5 expression (Figure 4J). However, in these experiments, cell growth was inhibited following *Raptor* KD (Supplementary Figure 4E), requiring that the amount of Ccl5 present in the medium be normalized to the total cell number.

Since MEK/ERK activation is also elevated following *NF1* loss [22], we analyzed Ccl5 expression following PD-0325901 (PD901, MEK inhibitor) treatment. In these experiments, MEK inhibition did not change Ccl5 expression (Supplementary Figure 5). Collectively, these experiments establish RAS-GAP/AKT/mTOR signaling as the primary mechanism underlying neurofibromin regulation of Ccl5 expression.

### Ccl5 controls M-GBM survival through CD44

**Figure 5:**
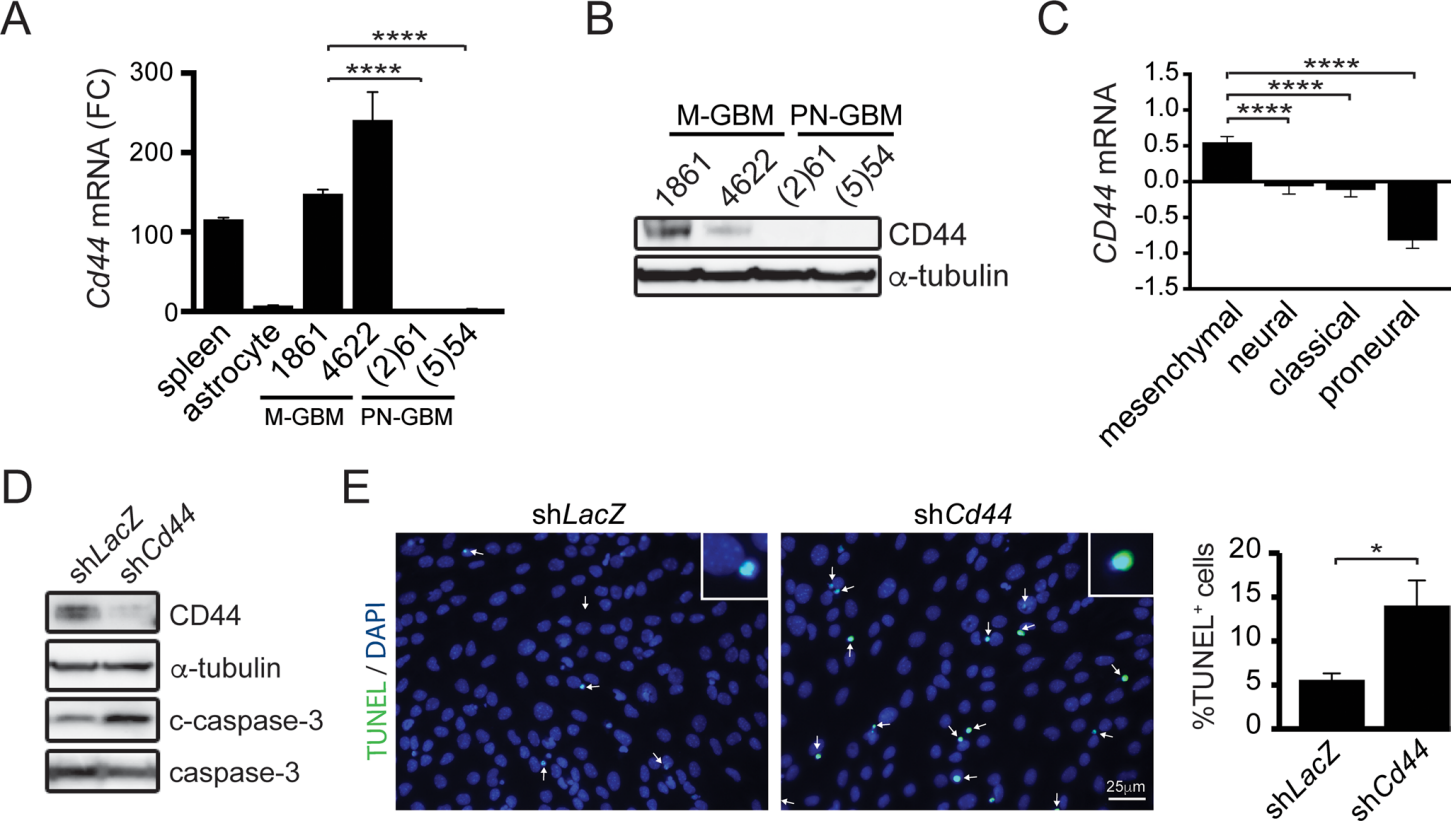
CD44 is required for M-GBM cell survival (**A**) qPCR analysis of *Cd44* mRNA in spleen (positive control), primary astrocytes, mesenchymal GBM (M-GBM) and proneural GBM (PN-GBM) cells reveals higher *Cd44* mRNA levels in M-GBM cells relative to PN-GBM cells and primary astrocytes. ****p < 0.0001. FC, fold change. (**B**) CD44 protein is detected by Western blot in M-GBM, but not in PN-GBM, cells. (**C**) *CD44* mRNA expression (Log2 Z-score) in TCGA GBM (provisional) samples segregated by molecular subgroup reveals that mesenchymal and proneural GBM express the highest and lowest *CD44* levels, respectively. ****p < 0.0001. (**D**) *Cd44* KD 1861 cells (sh*Cd44*) have increased cleaved caspase-3 (c-caspase-3) expression relative to control (sh*LacZ*) cells. (**E**) Increased %TUNEL^+^ cells (inset) are observed following *Cd44* KD (green; arrows). DAPI-stained nuclei are shown in blue. *p = 0.0288. Scale bar, 25 μm.

CCL5 functions by binding to G-protein coupled receptors, including CCR (C-C chemokine receptor) 1, 3 and 5. Surprisingly, *Ccr1*, *Ccr3* or *Ccr5* mRNA levels were nearly undetectable in M-GBM cells (Supplementary Figure 6), prompting us to investigate non-conventional CCL5 receptors (GPR75, CXCR4 and CD44) as potential binding partners [23, 24]. In these experiments, only *Cd44* mRNA was detected in 1861 and 4622 cells (Figure 5A), which also observed at the protein level (Figure 5B). Importantly, *CD44* mRNA expression in human GBM subtypes was highest in the mesenchymal group, and was lowest in the proneural group (Figure 5C).

**Figure 6:**
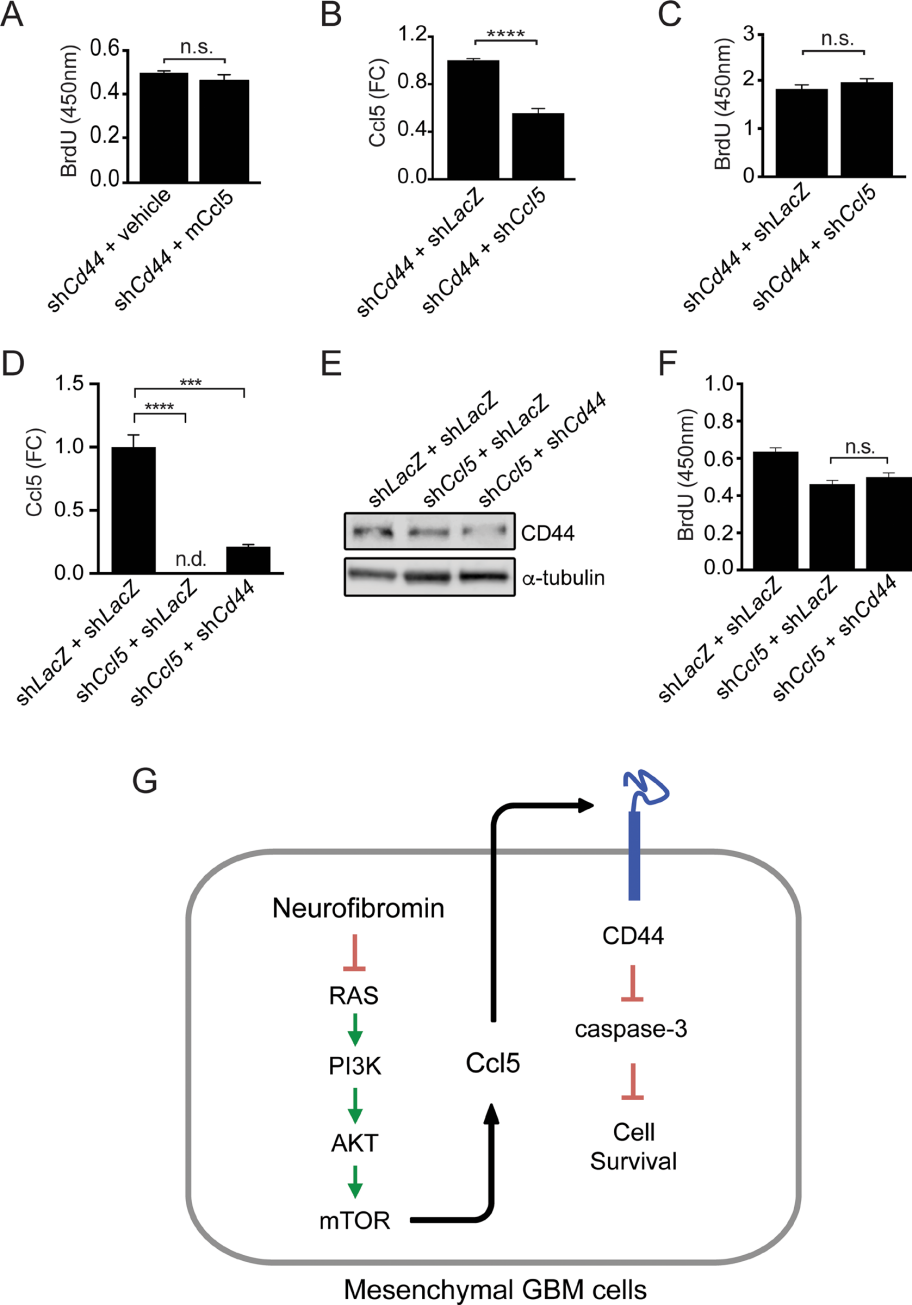
Ccl5 regulates M-GBM survival through CD44 (**A**) *Cd44* KD 1861 cells treated with vehicle (0.5% BSA in PBS) or mouse Ccl5 (mCcl5) show no difference in BrdU incorporation. n.s., not significant. (**B**) ELISA analysis shows that sh*Cd44* + sh*Ccl5* cells have reduced levels of secreted Ccl5 relative to controls (sh*Cd44* + sh*LacZ*). ****p < 0.0001. FC, fold change. (**C**) *Ccl5* KD in *Cd44* KD cells did not change BrdU incorporation. n.s., not significant. (**D**) ELISA analysis shows that *Ccl5* KD cells (sh*Ccl5* + sh*LacZ* and sh*Ccl5* + sh*Cd44*) have reduced levels of secreted Ccl5 relative to control cells (sh*LacZ* + sh*LacZ*). ****p < 0.0001. ***p = 0.0002. FC, fold change. n.d., not detected. (**E**) Western blotting demonstrates that *Cd44* KD cells (sh*Ccl5* + sh*Cd44*) have less CD44 protein relative to other cells (sh*LacZ* + sh*LacZ* and sh*Ccl5* + sh*LacZ*). (**F**) *Cd44* KD in *Ccl5* KD cells did not change BrdU incorporation. n.s., not significant. (**G**) Proposed model for Ccl5 regulation of M-GBM cell survival.

To determine whether CD44 was necessary for M-GBM cell survival, we reduced *Cd44* expression in 1861 cells using shRNA. Following *Cd44* KD, there was increased apoptosis (cleaved caspase-3 expression and %TUNEL^+^ cells) relative to controls (*LacZ* KD) (Figure 5D, 5E). In order to demonstrate that CD44 functions as the receptor for Ccl5 in 1861 cells, *Cd44* KD cells were treated with mCcl5. Following *Cd44* KD, mCcl5 could no longer increase 1861 cell growth (Figure 6A). Similarly, *Ccl5* KD in *Cd44* KD cells (Figure 6B) did not change cell growth (Figure 6C), establishing that the growth-promoting effects of Ccl5 require CD44 expression on M-GBM cells. In order to test whether other CD44 ligands function in a similar fashion as Ccl5, we reduced *Cd44* using shRNA in *Ccl5* KD cells (sh*Ccl5* + sh*Cd44*), and compared cell proliferation to that observed in *Ccl5* KD (sh*Ccl5*) and control (sh*LacZ*) cells. It should be noted that the sh*LacZ* and sh*Ccl5* cells were further infected with virus carrying the sh*LacZ* construct as an internal control for a second round of viral infection. We observed no further reduction of cell proliferation in *Ccl5* KD cells following *Cd44* knockdown (Figure 6D–6F), suggesting that this pro-survival pathway is specific to the interaction between Ccl5 and CD44, and is not regulated by other CD44 ligands. Taken together, these data support a model in which *Nf1* loss in M-GBM cells leads to hyperactivation of the AKT/mTOR pathway, which positively regulates Ccl5 expression and increases M-GBM cell survival through CD44 signaling (Figure 6G).

## DISCUSSION

Chemokines were originally described as key signaling molecules in cancer biology by virtue of their ability to recruit stromal cells (e.g., macrophages, endothelial cells) to the developing tumor [25, 26]. While this is one important function for these immune modulatory proteins, emerging evidence suggests that chemokines and cytokines may create autocrine growth regulatory circuits that facilitate tumor expansion. In this regard, CXCR2 signaling has been shown to regulate KRAS-induced pancreatic cancer growth [27], whereas IL-8 serves as an autocrine growth factor for malignant mesothelioma maintenance [28]. In the context of glioma, CXCL12/CXCL12-receptor (CXCR4/CXCR7) autoregulatory signaling has been demonstrated to be important for glioblastoma survival [8, 29, 30] and angiogenesis [31], while CX3CL1/CX3CR1 axis signaling in glioma cells controls glioma cell invasion [32].

The emergence of such autoregulatory chemokine/chemokine receptor loops relevant to glioma growth prompted us to examine a recently-identified paracrine signal (CCL5), produced by low-grade glioma-associated microglia [11], as a potential autocrine signaling molecule in high-grade glioma. While low-grade gliomas are dependent on growth-facilitatory signals from the tumor microenvironment, we hypothesize that their malignant counterparts achieve partial stromal independence by producing the very growth factors elaborated by non-neoplastic cells in low-grade gliomas. In this study, we demonstrate that M-GBM cells express high levels of CCL5 that provides an auto-stimulatory signal to increase cell survival. Similarly, in other studies, M-GBM cells express high levels of CXCL12, which forms another autoregulatory loop with its receptor CXCR4/CXCR7, to increase GBM cell proliferation [8, 29, 30]. In NF1-associated low-grade gliomas, CXCL12 also serves as a paracrine factor that drives the growth of neoplastic *Nf1*−/− astrocytes [33]. These findings support the idea that high-grade glioma cells achieve certain degrees of stromal independence by producing growth factors previously provided by non-neoplastic stromal cells in the low-grade glioma tumor microenvironment.

CCL5 is a potent chemokine operative in several types of cancers, including lymphoma, melanoma, prostate cancer and breast cancer [34]. However, while CCL5 function in glioma has not been fully elucidated, several reports have suggested that it might be an important chemokine in this setting. First, one of the main CCL5 receptors, CCR5, is expressed in U87 and U251 glioblastoma cell lines, such that its knockdown inhibits U87 growth in nude mice [35]. Second, CCL5 mRNA and protein expression are elevated in both tumor tissue and serum from patients with high-grade glioma [36]. Lastly, CCL5 is produced by microglia in the microenvironment of murine *Nf1* optic glioma, and is increased in low-grade gliomas (pilocytic astrocytoma) relative to non-neoplastic tissue [11]. In this latter study, CCL5 treatment increased optic nerve glial proliferation *in vitro*, whereas antibody-mediated CCL5 inhibition reduced optic glioma growth *in vivo*.

CCL5 typically functions by binding to its receptors CCR1, CCR3 and/or CCR5. Surprisingly these receptors were not detectable in M-GBM cells. Instead, M-GBM cells express the non-conventional CCL5 receptor, CD44, and use this receptor to maintain tumor cell survival. CD44 is a cell surface glycoprotein that can function as a co-receptor for extracellular matrix components, growth factors and chemokines. CD44 plays several critical roles in GBM and drives GBM cell invasion, proliferation and drug resistance. CD44 expression is increased in GBM compared to low-grade gliomas and high levels of CD44 are associated with worse survival of GBM patients [37]. Inhibiting CD44 function using monoclonal antibodies or RNA silencing reduced GBM growth *in vivo* and prolonged mouse survival [38, 39]. The interaction between CD44 and its ligands (e.g., hyaluronic acid and osteopontin) modulates multiple aspects of glioma biology. Activation of CD44 by hyaluronic acid was shown to increase glioma cell invasion [40–43]. In addition, osteopontin-CD44 interaction induces cleavage of the intracellular domain of CD44, increases a stem cell phenotype, and increases the growth of PN-GBM cells [44]. In this study, we found that the interaction between Ccl5 and CD44 enhances cell survival in M-GBM cells. This pro-survival effect is specific to Ccl5, and does not appear to involve other CD44 ligands, since *Cd44* knockdown in *Ccl5* KD cells does not further reduce cell growth. Our study expands our understanding of the potential mechanisms by which CD44 regulates GBM maintenance.

CCL5 employs CD44 as an alternative receptor to facilitate the entry of HIV virus [24], where it forms a complex with CD44 and Src kinases to activate ERK signaling. In our studies, ERK activity in M-GBM cells was not reduced following *Ccl5* KD (data not shown), suggesting that a distinct signaling pathway is activated following Ccl5 binding to CD44 in M-GBM cells. Previous studies have demonstrated increased cell survival following CD44 activation in several different cancers. For example, CD44 protects chronic lymphocytic leukemia cells from spontaneous and induced cell death by increasing expression of the MCL-1 pro-survival protein [45], while CD44 activation in breast cancer cells induces c-Jun mediated survival [46]. It has been suggested in GBM that CD44 also inhibits the Hippo-mediated apoptotic signaling pathway to achieve resistance to reactive oxygen species- or cytotoxic agent-induced stress [39]. Further work will be required to determine how CD44 regulates cell survival in M-GBM cells.

GBM tumors are genetically heterogeneous and can be divided into mesenchymal, classical, neural and proneural subtypes. Herein, we focused on studying the behavior of mesenchymal GBM since Ccl5 has the highest expression in this group. One common feature of the M-GBM subtype is *NF1* loss/mutation, which results in impaired neurofibromin RAS suppression. Consistent with this observation, we demonstrated that neurofibromin directly regulates CCL5 expression through RAS signaling. First, we show that ectopic expression of a functional, but not mutant, NF1-GAP-related domain reduced Ccl5 expression. Second, *Nf1* loss in primary astrocytes resulted in increased Ccl5 expression. Third, pharmacological inhibition of RAS downstream effector activation (AKT/mTOR) suppressed Ccl5 expression in M-GBM cells. Collectively, these results elucidate one mechanism by which Ccl5 expression is controlled by RAS.

The Ccl5-mediated autocrine circuit identified in this study may not apply to other GBM subtypes, given that they do not express high levels of either CCL5 or CD44. In fact, we have shown that treating murine PN-GBM cells with Ccl5 did not increase cell growth. While multiple signaling pathways contribute to GBM tumorigenesis, we report one in which a chemokine-driven autocrine pathway supports GBM cell survival. The regulation of chemokines by oncogenic signaling pathways suggests that other brain cancer-associated mutations might similarly modulate chemokine production. Further dissection of the chemokine/cytokine networks regulated by these tumor suppressor/oncogenic pathways might uncover unique immune modulatory programs with differential impact not only on tumor cell growth, but also on the stromal cell types in the tumor microenvironment.

Lastly, while Ccl5 silencing results in ~20% reduction in cell growth *in vitro*, there was a ~70% reduction in tumor cell growth following *Ccl5* KD *in vivo*. The greater cell survival benefit *in vivo* raises the possibility that CCL5 may not solely function in an autocrine manner, but may also recruit monocytes (macrophages/microglia) to the tumor and enhance tumor growth in a paracrine manner. However, we found no difference in the percent of intratumoral Iba1^+^ monocytes in mice bearing *Ccl5* KD versus control (*LacZ* KD) M-GBM cells (data not shown). Since it is possible that attenuated Ccl5 expression alters the functional status of tumor-associated macrophages/microglia, current studies are focused on identifying potential changes in the tumor microenvironment conferred by reduced glioblastoma tumor cell Ccl5 production *in vivo*.

In summary, we demonstrate, for the first time, that CCL5 functions in an autocrine growth-promoting circuit, and establish a new receptor responsible for CCL5 function in mesenchymal glioblastoma cells. We further show that key oncogenic signaling pathways, like RAS, operate to modulate chemokine expression. As such, these studies expand the role of chemokines in dictating glioma biology, and suggest that paracrine growth regulatory signals produced by non-neoplastic cells in the low-grade glioma microenvironment may be converted to autocrine tumor maintenance circuits in high-grade malignancies relevant to mesenchymal glioblastoma survival *in vitro* and *in vivo*.

## MATERIALS AND METHODS

### Human *CCL5* and *CD44* expression analysis

Expression values for *CD44* and *CCL5* (Glioblastoma, TCGA Provisional, mRNA Expression z-Scores) were obtained from the MSKCC computational biology cancer genomics portal (http://www.cbioportal.org), which contains annotated TCGA data [47, 48]. Tumor subtype classification was performed as previously described [15].

### Mice

4-week-old male C57BL/6 mice were purchased from Taconic, and used in accordance with an approved Animal Studies Committee protocol at Washington University.

### Cell culture

Mouse mesenchymal glioblastoma cells (1861 and 4622) were maintained as previously described [49], while mouse proneural glioblastoma cells ((2)61 and (5)54)) were derived from PDGFB-driven tumor-bearing mice [18] and grown in mouse neural stem cell medium (STEMCELL Technologies, 05700 and 05701), supplemented with 20 ng/ml hEGF (Sigma-Aldrich, E9644), 10 ng/ml hFGF (R&D systems, 233-FB-025) and 2 μg/ml heparin (STEMCELL Technologies, 07980). Wild-type and *Nf1*−/− primary astrocytes were generated and maintained as previously described [14, 50, 51]. Before performing qPCR, ELISA or Western blotting, all cells were cultured under the same condition for at least 24 hours.

### Lentiviral infection

pLKO.1 plasmids with shRNAs targeting *LacZ*, *Ccl5* (sh*Ccl5*-1 and sh*Ccl5*-2), and *Cd44* were obtained from the Genome Institute at Washington University, while sh*Raptor*-1 and sh*Raptor*-2 plasmids were purchased (Addgene, 21339 and 21340) (Supplementary Table 1). shRNAs were prepared and cells infected as previously reported [49]. MSCV-NF1-GRD-WT and mutant control constructs [19] were transfected into 1861 cells using X-tremeGENE 9 DNA transfection reagents (Roche Life Science, XTG9-RO) according the manufacturer's protocol.

### Pharmacologic treatments

1861 cells were treated with 0.4 μM ZSTK474 (Selleckchem, S1072), 1 μM MK2206 (Selleckchem, S1078), 5 nM rapamycin (Selleckchem, S1039), 1 nM PD-0325901 (PD901; Selleckchem, S1036) or DSMO (vehicle control, Sigma-Aldrich, D8418) for 4 hours at 37°C.

### BrdU incorporation

Cells were plated in 96-well plates, and treated with 250 ng/mL mCcl5 (R&D systems, 478-MR-025) or 0.5 % BSA in PBS (vehicle control) for 4 hours at 37°C. BrdU was added to the cell culture 2 hours before fixing the cells and performing the BrdU ELISA assay (Roche Life Science, 11647229001) according the manufacturer's protocol.

### Terminal deoxynucleotidyl transferase dUTP nick end labeling (TUNEL)

Cells maintained in 0.5% fetal bovine serum for 24 hours were fixed with 4% PFA, and TUNEL^+^ cells detected using the fluorescence-based *in situ* cell death detection kit (Roche Life Science, 11684795910). The number of TUNEL^+^ cells was normalized to the total cell number (DAPI^+^ nuclei) using NIH ImageJ software.

### Western blotting

1861 cells expressing shRNAs were maintained in 0.5% fetal bovine serum for 24 hours prior to lysis and Western blotting with appropriate primary antibodies (Supplementary Table 2) [14, 52]. Quantification of western blots were performed using ImageStudio (LI-COR). AKT and S6 activities were quantified as signal intensity of phospho-proteins versus signal intensity of total proteins.

### Mouse chemokine enzyme-linked immunosorbent assay (ELISA)

Cells were serum starved for 24 hours, and the culture medium collected for mouse Ccl5 ELISA (R&D systems, MMR00). The amount of secreted Ccl5 determined by ELISA was normalized to total cell number.

### Quantitative real-time PCR

qPCR was performed as described previously [11] using validated primers (Supplementary Table 3).

### Intracranial injections

5 × 10^4^ 1861 cells were implanted into the striatum of 4-week-old C57BL/6 mice as previously described [49]. Mice were monitored daily, and euthanized when moribund. Kaplan-Meier survival curves were generated, and differences between control (sh*LacZ*) and *Ccl5* KD (sh*Ccl5*-1 and sh*Ccl5*-2) groups analyzed using the Log-rank test. For mCcl5 ELISA and Western blotting of the implant, tissue around the tumor implantation site was dissected 6-weeks post-implantation, followed by lysing the tissue as described previously [14, 52].

### Immunohistochemistry

Implanted mouse brains were fixed at the time of death or deemed moribund by the veterinary staff, embedded in paraffin and processed as described previously [53], followed by performing immunohistochemistry using primary and secondary antibodies (Supplementary Table 2). Ccl5 signal was amplified using tyramide signal amplification as described previously [11].

### Statistical analyses

Data analysis was performed using Prism GraphPad. Unpaired two-tailed Student's *t* tests were used to determine the difference between two groups. All data were presented as mean values with standard error of mean (SEM). All *in vitro* experiments were repeated at least three times with similar results.

## SUPPLEMENTARY MATERIALS FIGURES AND TABLES


