# Supplementary material for: Ccl5 establishes an autocrine high-grade glioma growth regulatory circuit critical for mesenchymal glioblastoma survival
Source: Oncotarget. 2017 Mar 23;8(20):32977–89. doi: 10.18632/oncotarget.16516 (PMC5464843; doi:10.18632/oncotarget.16516)
Supplement: Supplementary file 1 [file oncotarget-08-32977-s001.pdf]

Ccl5 establishes an autocrine high-grade glioma growth regulatory circuit critical for mesenchymal glioblastoma survival

Supplementary Materials

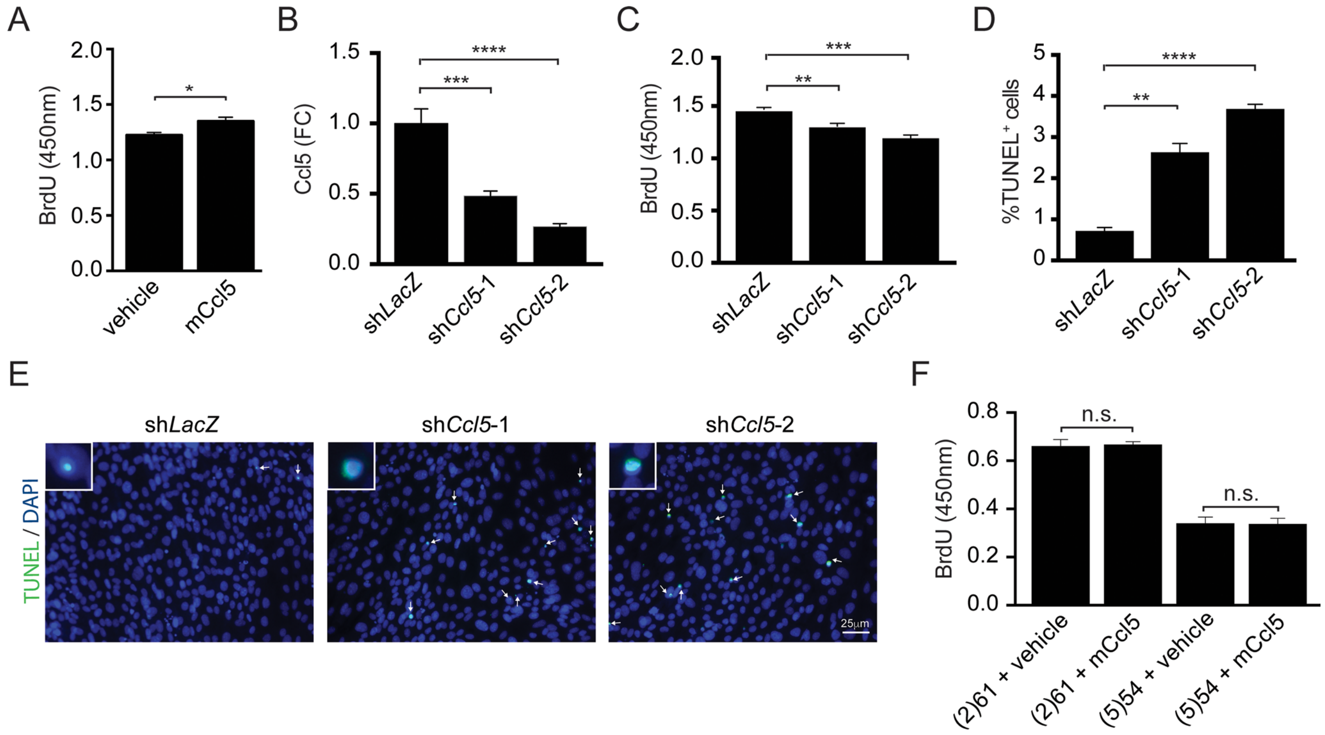

Supplementary Figure 1: Ccl5 increases 4622 M-GBM cell survival. (**A**) mCcl5 increased 4622 cell BrdU incorporation relative to vehicle. *$p = 0.0111$. (**B**) ELISA of 4622 cell culture medium shows that *Ccl5* KD cells (sh*Ccl5*-1, sh*Ccl5*-2) secrete less Ccl5 relative to controls (shLacZ). ***$p = 0.0003$. ****$p < 0.0001$. FC, fold change. (**C**) *Ccl5* KD cells exhibit reduced BrdU incorporation. **$p = 0.0057$. ***$p = 0.0002$. (**D**, **E**) Increased %TUNEL+ cells (inset) are observed following *Ccl5* KD (green; arrows). DAPI-stained nuclei are shown in blue. **$p = 0.0011$; ****$p < 0.0001$. Scale bar, 25 μm. (**F**) PN-GBM cells ((2)61 and (5)54)) treated with vehicle (0.5% BSA in PBS) or mCcl5 do not show difference in cell growth. n.s., not significant.

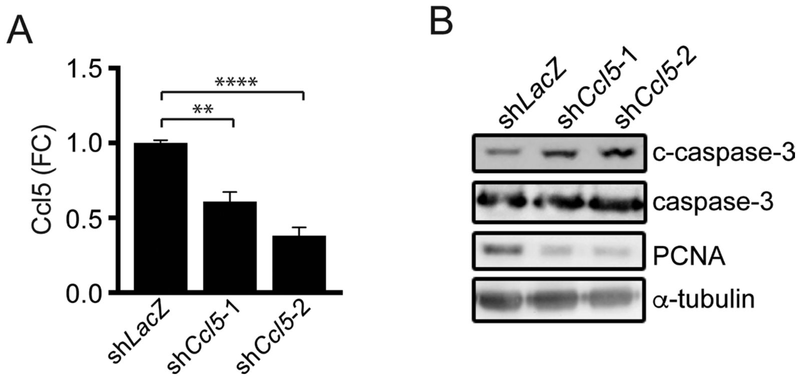

Supplementary Figure 2: *Ccl5* knockdown induces M-GBM cell death *in vivo*. (**A**) *Ccl5* KD (sh*Ccl5*-1, sh*Ccl5*-2) and control (sh*LacZ*) 1861 cells were implanted into the striata of C57BL/6 mice, and the tumor-containing regions dissected 6 weeks later for analysis. Ccl5 ELISA shows decreased Ccl5 levels in the *Ccl5* KD groups relative to controls. $**p = 0.0012$; $****p < 0.0001$. (**B**) Western blotting demonstrates increased cleaved caspase-3 and decreased proliferating cell nuclear antigen (PCNA) expression in *Ccl5* KD groups relative to controls.

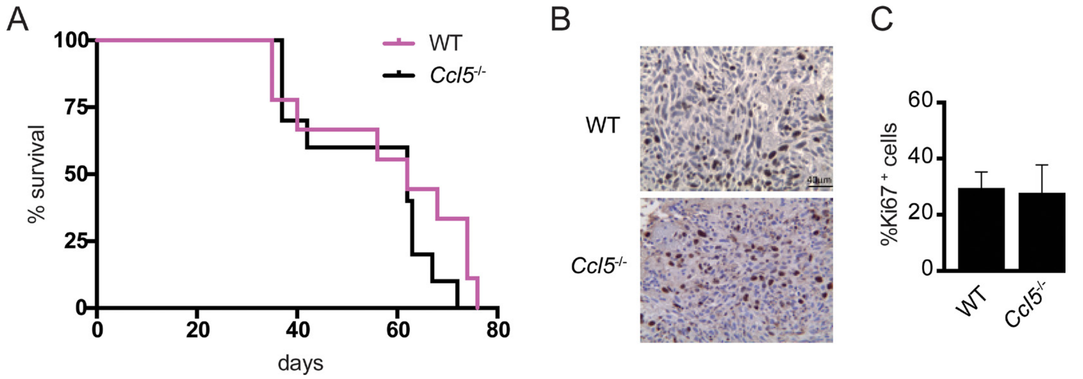

Supplementary Figure 3: Ccl5 secreted by non-neoplastic cells does not contribute to M-GBM growth *in vivo*. (**A**) 1861 cells were implanted into the striata of wild-type and *Ccl5−/−* mice. Kaplan-Meier survival curves demonstrate no difference between the wild-type and *Ccl5−/−* groups ($p = 0.231$). Each group contains 9-10 mice. (**B**, **C**) Immunohistochemistry demonstrates no difference in the percentage of Ki67+ cells between the wild-type and *Ccl5−/−* groups ($p = 0.8756$).

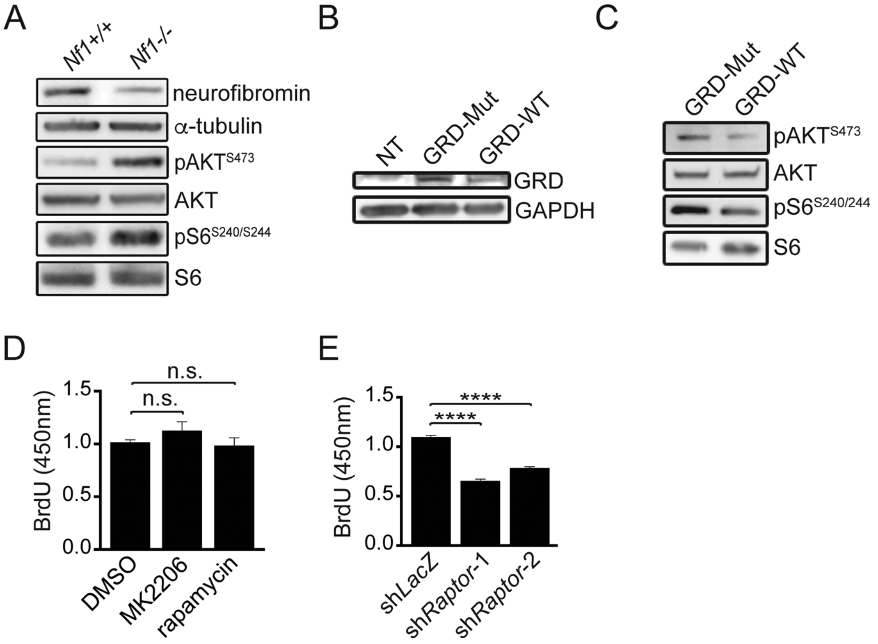

Supplementary Figure 4: Activation of the AKT/mTOR pathway following *Nf1* loss. (**A**) *Nf1−/−* primary astrocytes exhibit increased AKT/mTOR pathway activation relative to wild-type (*Nf1+/+*) astrocytes. (**B**) Exogenous expression of wild-type NF1-GRD (GRD-WT) and mutant R1276P-GRD (GRD-Mut) in 1861 cells. NT, no transfection. (**C**) GRD-WT expression reduced AKT/mTOR activity relative to GRD-Mut expression. (**D**) MK2206 or rapamycin treatment of 1861 cells did not alter BrdU incorporation. n.s., not significant. (**E**) *Raptor* KD decreased 1861 cell growth (BrdU incorporation). ****$p < 0.0001$.

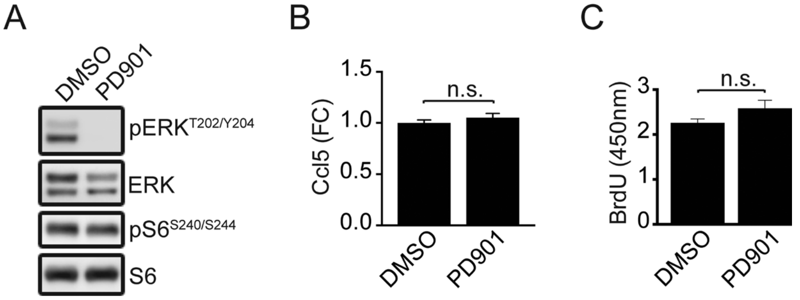

Supplementary Figure 5: Ccl5 expression is not regulated by MEK/ERK pathway. (**A**) PD901 (MEK inhibitor) treatment of 1861 cells suppressed ERK, but not mTOR, activity. DMSO, vehicle. (**B**) ELISA shows no change in secreted Ccl5 following PD901 treatment. n.s. not significant. FC, fold change. (**C**) PD901 treatment of 1861 cells did not change BrdU incorporation. n.s., not significant.

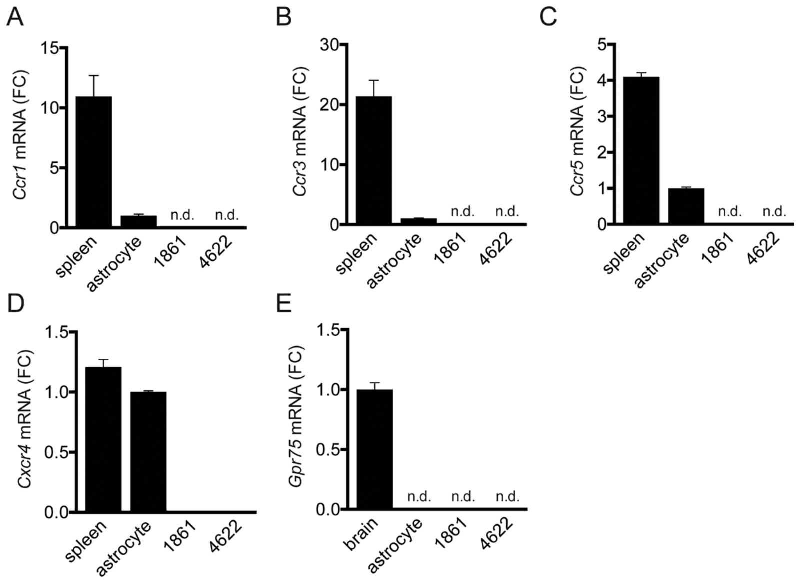

Supplementary Figure 6: Ccl5 receptor expression in M-GBM cells. qPCR analysis of Ccl5 receptor expression, including *Ccr1* (**A**), *Ccr3* (**B**), *Ccr5* (**C**), *Cxcr4* (**D**) and *Gpr75* (**E**), in spleen/brain (positive controls), primary astrocytes, 1861 cells and 4622 cells. All of these receptors were below the level of detectability in 1861 or 4622 cells. FC, fold change. n.d., not detected.

Supplementary Table 1: shRNA constructs used

Construct	Target sequence
sh*Ccl5-1*	ccagagaagaagtgggttcaa
sh*Ccl5-2*	cgtgtttgtcactcgaaggaa
sh*Cd44*	cctcccactatgacacatatt
sh*LacZ*	cccgtcagtatcggcggaatt
sh*Raptor-1*	cctcatcgtcaagtccttcaactc
sh*Raptor-2*	gcccgagtctgtgaatgtaatctc

Supplementaru Table 2: Antibodies used

Antibody	Host	Source	Dilution
AKT (WB)	Rabbit	Cell Signaling, 9272	1:1000
α-tubulin (WB)	Mouse	Sigma, T9026	1:20,000
anti-mouse HRP (IHC)	-	Vector, BA-9200	1:200
anti-mouse HRP (WB)	-	R&D, 7076	1:5000
anti-rabbit HRP (WB)	-	R&D, 7074	1:5000
caspase-3 (WB)	Rabbit	Cell Signaling, 9665	1:1000
Ccl5 (IF)	Rabbit	LSBio, LS-C104689<br>LS-C104689	1:200
CD44 (WB)	Rabbit	Abcam, ab137820	1:500
cleaved caspase-3 (WB)	Rabbit	Cell Signaling, 9664	1:1000
ERK (WB)	Rabbit	Cell Signaling, 9102	1:1000
Ki67 (IHC)	Mouse	BD Pharmingen, 550609	1:500
neurofibromin (WB)	Rabbit	Santa Cruz, SC-67	1:200
PCNA (WB)	Mouse	Abcam, ab29	1:1000
phospho-AKT^{S473} (WB)	Rabbit	Cell Signaling, 4060	1:200
phospho-ERK$^{T202/Y204}$ (WB)	Rabbit	Cell Signaling, 9101	1:1000
phospho-S6$^{S240/S244}$ (WB)	Rabbit	Cell Signaling, 2215	1:10,000
S6 (WB)	Rabbit	Cell Signaling, 2217	1:5000

WB: Western Blot; IHC: immunohistochemistry; IF: immunofluorescence; HRP: horseradish peroxidase.

Supplementary Table 3: qRT-PCR primers used

Target gene	Forward primer sequence	Reverse primer sequence
Ccl5	aatcttgcagtcgtgtttgtca	agctcatctccaaatagttgatgt
Ccr1	taggttgggaccttgaaccttg	aaagacagtgagtctgtgtttcc
Ccr3	ctactggactcataaaggacttagc	ggtgcccactcatattcatagg
Ccr5	atccgttccccctacaagaga	tggcagggtgctgacatac
Cd44	ggatgaatcctcggaattacca	gctttcaacagtaccttaccca
Cxcr4	catggaaccgatcagtgtgagt	tggtgggcaggaagatcctat
Gpr75	tcaggatctcagctcacaga	agatagggtcactactgcga
